# Surgical management of papillary thyroid carcinoma coexisting with Hashimoto’s disease: a single-center retrospective cohort study

**DOI:** 10.3389/fendo.2024.1383945

**Published:** 2024-08-07

**Authors:** Dongdong Zhang, Jixiang Wu, Lin Chen

**Affiliations:** Department of Gastrointestinal Surgery, International Hospital, Peking University, Beijing, China

**Keywords:** papillary thyroid cancer, Hashimoto disease, low-risk, radical surgery, central neck dissection

## Abstract

**Background:**

The mechanism and impact of Hashimoto’s disease (HT) in patients with papillary thyroid carcinoma (PTC) remains a subject of ongoing debate. The optimal extent of thyroid resection is also controversial in cases of low-risk PTC.

**Objective:**

To investigate the clinical outcomes and prognoses associated with different extents of surgical resection in patients diagnosed with PTC coexisting with HT.

**Methods:**

We retrospectively analyzed data on the clinical features and treatment outcomes of patients with PTC concomitant with HT who underwent lobectomy with isthmusectomy and those who underwent total thyroidectomy at Peking University International Hospital between December 2014 and August 2023.

**Results:**

Twenty-one patients in group A underwent lobectomy with isthmusectomy and prophylactic central neck dissection, whereas twenty patients in group B underwent total thyroidectomy with prophylactic central lymph node (LN) dissection, except one who did not undergo LN dissection. Group A demonstrated shorter surgery time (105.75 min ± 29.35 vs. 158.81 min ± 42.01, p = 0.000), higher parathyroid hormone (PTH) levels on postoperative day 1 [26.96 pg/ml (20.25, 35.45) vs. 9.01 pg/ml (2.48, 10.93), p = 0.000] and a shorter postoperative hospital stay [2.95 d (2.0, 4.0) vs. 4.02 d (3.0, 5.0), p = 0.008] than those of group B, with statistically significant differences. Both groups exhibited similar recovery patterns in terms of PTH [32.10 pg/ml (22.05, 46.50) vs. 20.47 pg/ml (9.43, 34.03), p = 0.192] and serum calcium (2.37 mmol/L ± 0.06 vs. 2.29 mmol/L ± 0.19, p = 0.409) after 1 montsh following the surgery. According to the Kaplan-Meier curves, no significant difference in the 5-year disease-free survival rates were observed between patients in group A (100%) and group B (97.1%) (Log rank test: p = 0.420, Breslow test: p = 0.420).

**Conclusion:**

Lobectomy with isthmusectomy and prophylactic central neck dissection is a safe and feasible treatment option for patients with low-risk PTC coexisting with HT.

**Clinical trial registration:**

http://www.chictr.org.cn, identifier ChiCTR2300079115.

## 1 Introduction

Papillary thyroid carcinoma (PTC) is the most common endocrine malignancy, and its incidence has been rapidly rising over the past decade (1, 2). It is classified as a highly differentiated cancer with the most favorable prognosis among all types of thyroid cancers (1). Hashimoto’s thyroiditis (HT), also called chronic lymphocytic thyroiditis (CLT), is a common autoimmune endocrine disorder, responsible for most cases of hypothyroidism in areas with adequate iodine intake (3). Epidemiologic studies have reported a mean coexistence rate between HT and PTC of approximately 23% (range, 5%-85%) (4).

According to the National Comprehensive Cancer Network (NCCN) clinical practice guideline for thyroid cancer, indications for total thyroidectomy or lobectomy in patients with papillary carcinoma necessitates the presence of the following criteria: 1, no prior radiation exposure; 2, no distant metastases; 3, no lateral cervical lymph node (LN) metastases; 4, no extrathyroidal extension; and 5, presence of tumor of 1– 4 cm in diameter (5). Less aggressive management is associated with less complications. Notably, total thyroidectomy carries the risk of recurrent laryngeal nerve injury (2.5%, bilateral in rare cases) and temporary or permanent hypoparathyroidism (8.1%) (6). The risk (even when performed by high-volume surgeons) is almost twice that of lobectomy alone, and postoperative complications are generally more likely when performed by low-volume surgeons (7). The use of prophylactic central neck dissection for low-risk tumors (T1b–T2, N0) varies from center to center. Evidence of its effect on recurrence-free survival is conflicting, and no high-level evidence supporting or refuting its usefulness for low-risk tumors is available (8). Some retrospective studies have suggested HT as a protective factor against the risk of recurrence differentiated thyroid carcinoma (DTC) in patients with *BRAF* -wild type carcinomas (9). Current American Thyroid Association (ATA) guidelines specify *BRAF* mutation as a parameter associated with a higher risk of recurrence. However, the guidelines do not specify or highlight the potential role of HT as a protective factor (10).

We retrospectively analyzed the clinical data of patients diagnosed with PTC and HT, aiming to explore the clinical outcomes and prognoses associated with different extents of surgical removal to provide enhanced guidance for clinical practice in this domain.

## 2 Materials and methods

### 2.1 Study population

In this retrospective study, we collected clinical data of patients diagnosed with PTC and HT who underwent surgical procedures at Peking University International Hospital between December 2014 and August 2023. The collection and subsequent analysis of patients’ data was duly approved by the Biomedical Ethics Committee of Peking University International Hospital under No. 2023-KY-0093-01.

### 2.2 Methods

#### 2.2.1 Study design

This was a retrospective cohort study, aimed at the preliminary analysis and exploration of surgical outcomes, recent complications, and long-term prognosis in patients with PTC concomitant HT who underwent lobectomy with isthmusectomy and those who underwent total thyroidectomy. According to NCCN guideline, the extent of thyroidectomy was individualized and done in consultation with the patient diagnosed for low-risk PTC coexisting with HT.

#### 2.2.2 Inclusion and exclusion criteria

The inclusion criteria were as follows: 1) pathologically confirmed TNM stage T1-2 papillary thyroid carcinoma with coexisting Hashimoto’s disease; 2) having underwent standard radical surgery for thyroid cancer; 3) having underwent limited surgical intervention.

The exclusion criteria were as follows: 1) presenting with distant metastasis; 2) histopathological findings including follicular carcinoma, medullary carcinoma, poorly differentiated carcinoma, or undifferentiated carcinoma; 3) coexistence with diffuse toxic goiter, acute suppurative thyroiditis, or subacute thyroiditis; 4) having initially underwent regular follow-up surveillance and/or interventions such as radiofrequency ablation or intra-iodine-131 radiation therapy.

#### 2.2.3 Surgical methods

**Figure 1:**
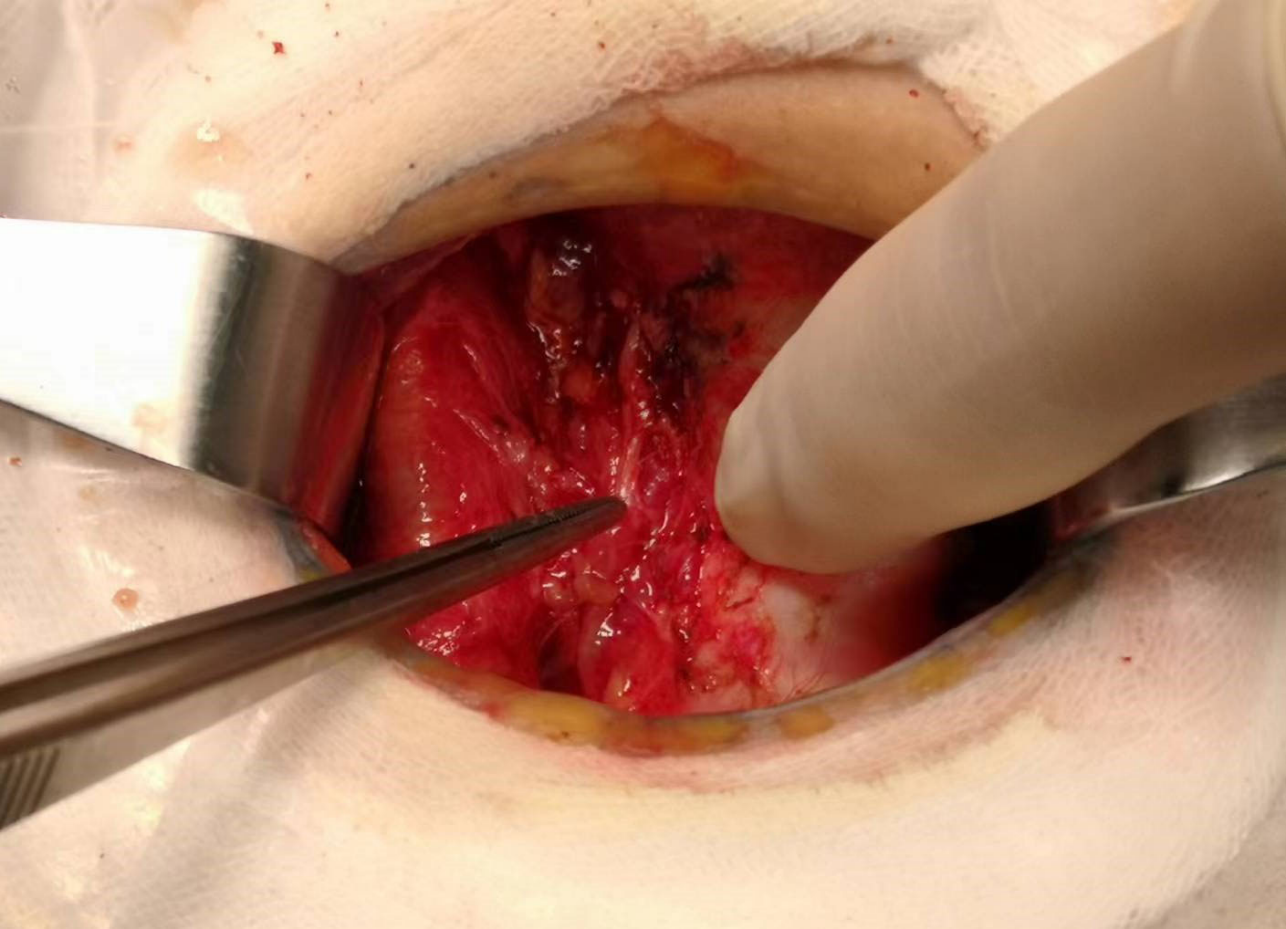
Right recurrent laryngeal nerve.

**Figure 2:**
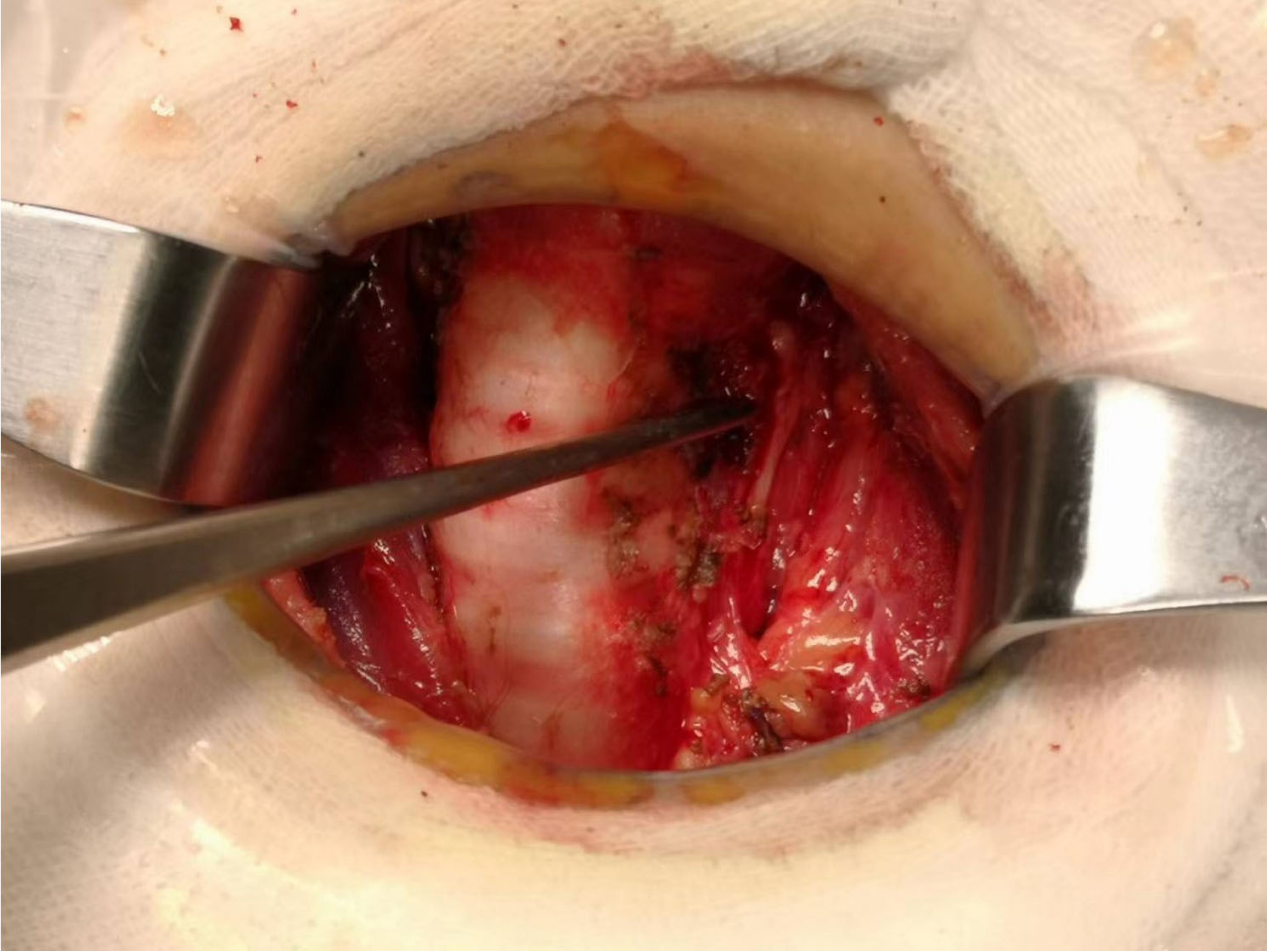
Left recurrent laryngeal nerve.

**Figure 3:**
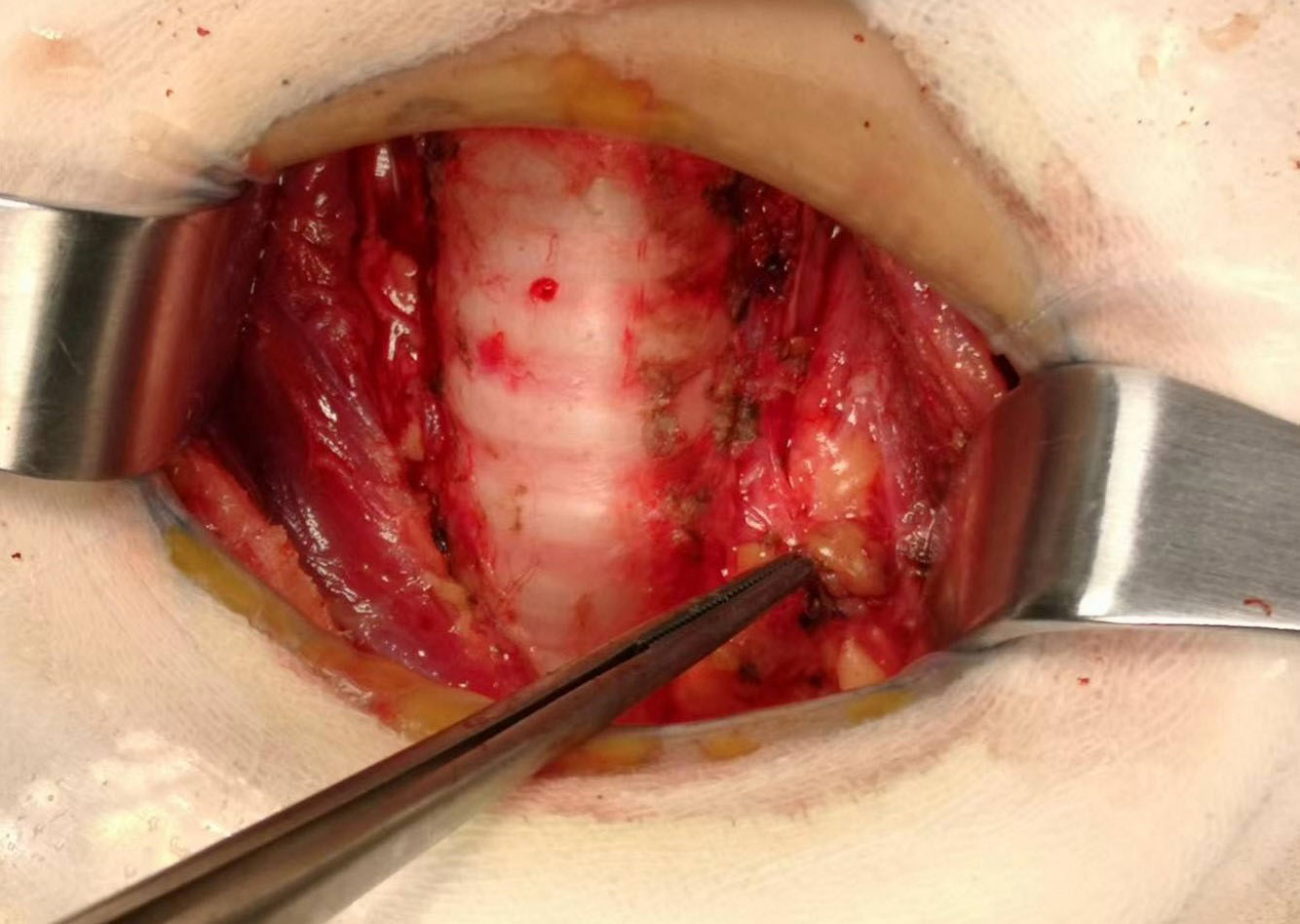
The parathyroid gland preserved *in situ*.

**Figure 4:**
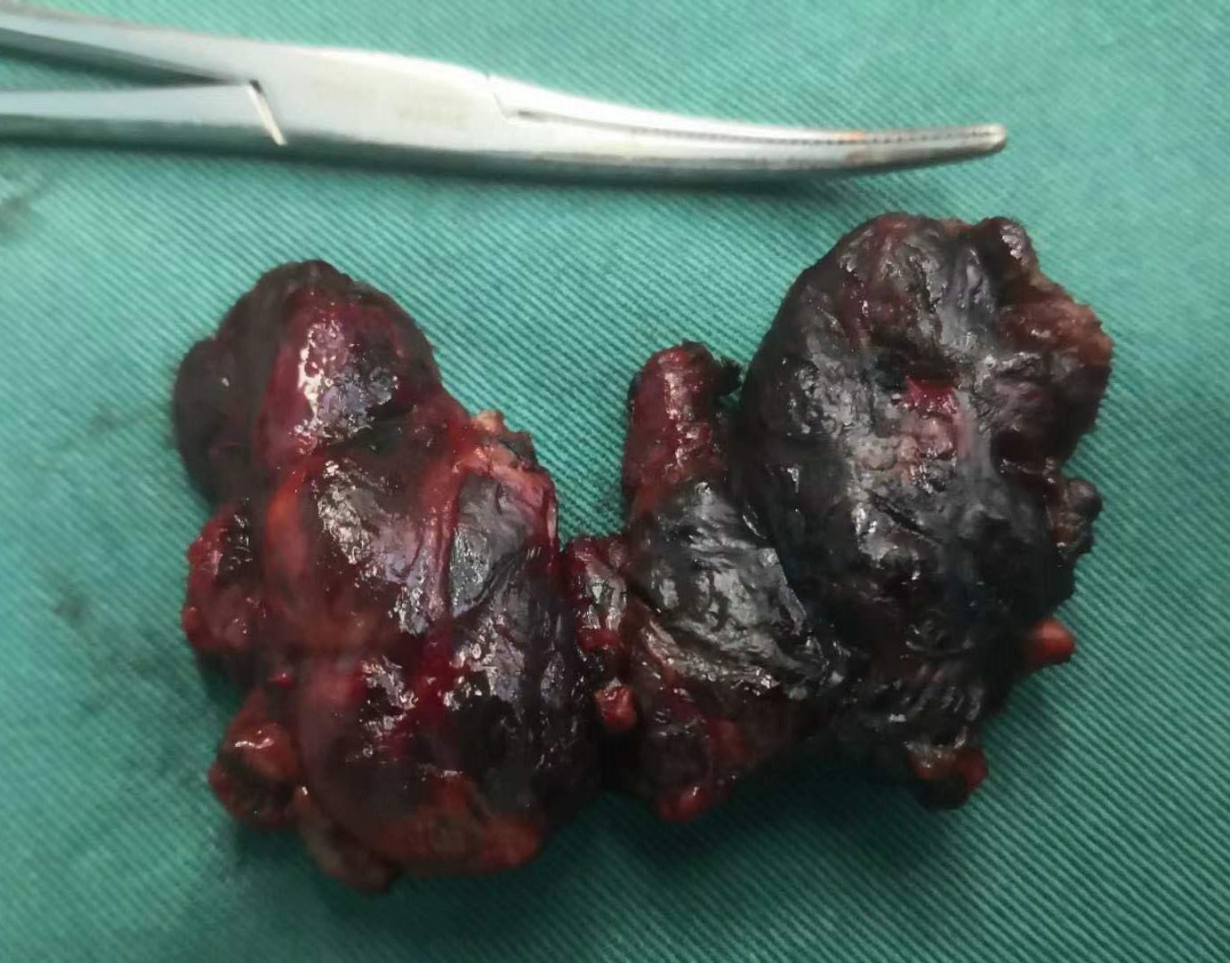
Gross specimen of bilateral thyroid and isthmus (The carbon nanoparticles are administered intrathyroidally).

Total thyroidectomy: The process involved careful dissection, ligation, and cutting of the branches of superior and inferior blood vessels of the thyroid poles, as well as the middle thyroid vein, in close proximity to the thyroid gland. The anatomical dissection revealed the recurrent laryngeal nerve; intraoperative nerve monitoring was used to examine signals from the recurrent laryngeal nerve, external branch of the superior laryngeal nerve, and vagus nerve. Both bilateral thyroid glands and isthmus tissue was completely resected. Negative staining technology with carbon nanoparticles was utilized to preserve the parathyroid gland *in situ*, and intraoperative autogenous parathyroid transplantation was conducted when the gland was inadvertent removed (Parathyroid glands were identified using a test paper). The images depicted in **Figures 1**–**4** illustrate the anatomical changes after surgery.

Lobectomy with isthmusectomy: Unilateral thyroid gland and isthmus tissue was radically resected according to the procedure of total thyroidectomy. Attention was also given to the protection of the parathyroid glands and nerves.

**Figure 5:**
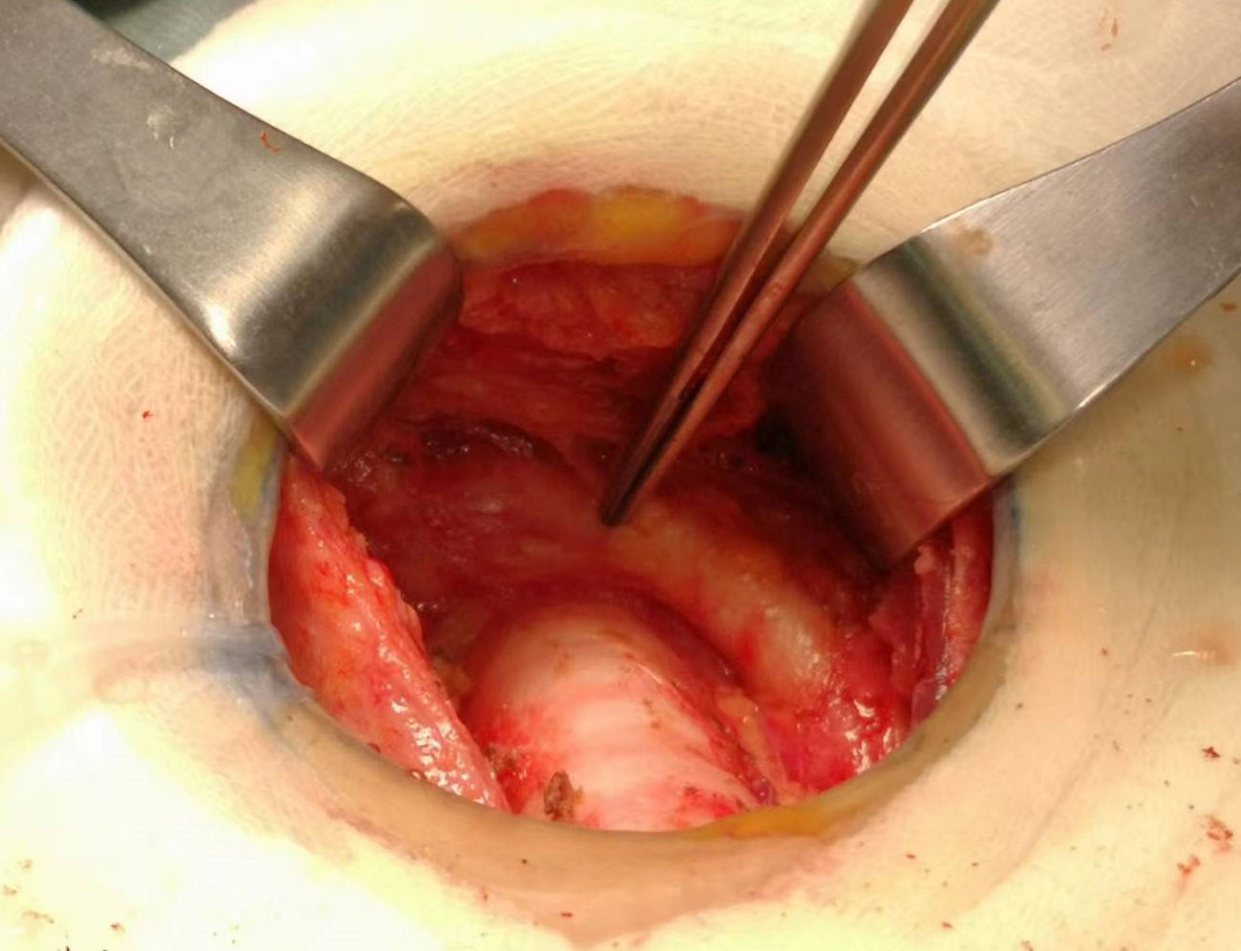
The postoperative manifestations of pretracheal lymph node dissection (the right innominate artery indicated by tweezer).

Prophylactic central neck dissection: The prelaryngeal nodes were removed superiorly, and the pretracheal nodes were removed inferiorly up to the innominate artery. Additionally, the paratracheal LNs were excised inferiorly from the cricoid cartilage to the right innominate artery and to the axial plane on the left where it intersects with the trachea (11). The operative area after dissection of the pretracheal LNs is depicted in **Figure 5**.

#### 2.2.4 Observation indicators

(1) Laboratory examination: Thyroid-stimulating hormone (TSH), parathyroid hormone (PTH), non-stimulated serum thyroglobulin (ns-Tg), thyroglobulin antibody (TG-Ab), thyroid peroxidase antibody (TPO-Ab), serum calcium.

(2) Surgical treatment: Lesion location (unilateral or bilateral, multifocality or not), Lesion diameter, surgical approach, operative time, postoperative complications, and pathological characteristics.

#### 2.2.5 Follow up methods

Patients were followed up through outpatient visits. Levels of thyroid hormone, parathyroid hormone, and blood calcium were assessed at 1 month post-surgery once a steady blood concentration of levothyroxine was established within the body. Follow-up was conducted every 3–6 months within 2 years of surgery, and annually thereafter. Follow ups encompassed physical examinations, assessment of TSH, PTH, ns-Tg, serum calcium levels and color ultrasound examinations. The endpoints of follow-up were tumor recurrence, metastasis, or patient death. The follow-up process was concluded by December 2023.

#### 2.2.6 Statistical methods

Descriptive analysis was performed using IBM SPSS Statistics for Windows, version 26.0 (International Business Machines Corporation; 1 New Orchard Road, Armonk, New York 10504-1722, United States). Continuous variables, including clinicopathologic characteristics, were compared across the groups using single-sample Kolmogorov-Smirnov test in order to assess their conformity with the normal distribution. Subsequently, an independent sample T-test was conducted. If the continuous variables did not adhere to a normal distribution, the Mann-Whitney test was used as a non-parametric alternative. The median and interquartile range were used to describe its characteristics. For categorical variables, the Fisher exact test was employed. Kaplan-Meier survival curves and log-rank tests were used censoring patients at the time of last follow-up. Significance levels were interpreted as two-sided with a p < 0.05.

## 3 Results

A total of 42 patients (33 female and 9 male) were included in the analysis. In group A (17 female and 4 male), 21 patients underwent lobectomy with isthmusectomy and prophylactic central neck dissection. In group B (16 female and 5 male), 20 patients underwent total thyroidectomy along with prophylactic central LN dissection, except one who did not undergo prophylactic central LN dissection. The mean age of patients in group A (44.39 years ± 11.26) was comparable to that of group B (47.18 years ± 10.89) (p = 0.407). The two groups did not exhibit any statistically significant disparities in terms of tumor staging (p = 0.931) and lesion type (p = 0.495). The baseline characteristics of the patients in the two groups are presented in **Table 1**.

**Table 1:** Comparison clinical features between patients with PTC concomitant HT who underwent lobectomy + isthmusectomy (Group A) and those who completed total thyroidectomy (Group B).

General Information	Data		
	Group A	Group B	Value P
Gender (Male/Female)	4/17	5/16	0.707
Age (years, $\bar{x}$ ± s)	44.39 ± 11.26	47.18 ± 10.89	0.407
BMI (kg/m^2^, $\bar{x}$ ± s)	25.86 ± 2.50	25.69 ± 4.72	0.101
Tumor Staging [cases]			
pT1a (tumor ≤1cm)	15	25	0.931
pT1b (tumor >1 cm but ≤2 cm)	6	9	
pT2 (tumor >2 cm but ≤4 cm)	1	1	
Lesion Type [cases]			
Unilateral and unifocal	16	14	0.495
Unilateral and multifocal	5	7	
Preoperative TSH level (uIU/ml, $\bar{x}$ ± s)	2.28 ± 1.53	2.92 ± 1.54	0.199
Preoperative TPOAb level [IU/ml, M (Q1, Q3)]	199.8 (11.3, 424.4)	66.6 (15.8, 167.4)	0.240
Preoperative TgAb level [IU/ml, M (Q1, Q3)]	200.6 (84.7, 392.6)	147.4 (94.4, 442.1)	0.908
Preoperative Tg level [μg/L, M (Q1, Q3)]	3.74(0.43, 11.95)	1.49(0.67, 14.91)	0.897
Preoperative PTH level (pg/ml, $\bar{x}$ ± s)	41.56 ± 10.52	40.02 ± 14.50	0.751
Preoperative serum calcium level (mmol/L, $\bar{x}$ ± s)	2.30 ± 0.13	2.31 ± 0.13	0.835
Prophylactic central neck dissection [cases]			
YES	21	20	0.311
NO	0	1	
Lymph node metastasis [cases]			
YES	4	5	0.707
NO	17	16	

Compared to group B, group A exhibited less surgery time (105.75 min ± 29.35 vs. 158.81 min ± 42.01, p = 0.000), higher PTH levels on postoperative day 1 [26.96 pg/ml (20.25, 35.45) vs. 9.01 pg/ml (2.48, 10.93), p = 0.000], and shorter postoperative hospital stay [2.95 d (2.0, 4.0) vs. 4.02 d (3.0, 5.0), p = 0.008] with statistically significant differences. Additionally, group A had lesser intraoperative bleeding volume [14 ml (10, 20) vs. 15 ml (10, 20), p = 0.740] and higher serum calcium levels on postoperative day 1 (2.19 mmol/L ± 0.12 vs. 2.12 mmol/L ± 0.21, p = 0.226) with no significant disparity statistically.

After 1 month post-surgery, both groups exhibited recovery in PTH [32.10 pg/ml (22.05, 46.50) vs. 20.47 pg/ml (9.43, 34.03), p = 0.192] and serum calcium (2.37 mmol/L ± 0.06 vs. 2.29 mmol/L ± 0.19, p = 0.409) levels, with group A demonstrating higher values than those of group B; however, this difference did not reach statistical significance. Additionally, no significant difference in TSH suppression was observed between the two groups at 1 month, 6 months, and 1 year after surgery and ns-Tg level in the first year after surgery. In group B, two patients exhibited a postoperative pathological subtype characterized by tall cell type, one patient experienced postoperative incision infection. It is noteworthy that, due to the surgeon’s expertise in intraoperative neuromonitoring and negative staining technology with carbon nanoparticles, only one patient experienced permanent hypoparathyroidism, with no instances of permanent nerve damage were observed. The treatment outcomes of the patients in the two groups are presented in **Table 2**.

**Table 2:** The treatment outcomes of patients with PTC concomitant HT who underwent lobectomy + isthmusectomy (Group A) and those who completed total thyroidectomy (Group B).

Surgical-related information	Data		
	Group A	Group B	Value P
Surgery time (minutes, $\bar{x}$ ± s)	105.75 ± 29.35	158.81 ± 42.01	*0.000*
Intraoperative bleeding volume [ml, M (Q1, Q3)]	14 (10, 20)	15 (10, 20)	0.740
Postoperative hospital stays [days, M (Q1, Q3)]	2.95 (2.0, 4.0)	4.02 (3.0, 5.0)	*0.008*
PTH on postoperative day one [pg/ml, M (Q1, Q3)]	26.96 (20.25, 35.45)	9.01 (2.48, 10.93)	*0.000*
Serum calcium on postoperative day one (mmol/L, $\bar{x}$ ± s)	2.19 ± 0.12	2.12 ± 0.21	0.226
TSH in the first month after surgery [cases]			
≤ 2 uIU/ml	16	12	0.071
> 2 uIU/ml	5	9	
ns-Tg in the first month after surgery [cases]			
< 0.04 μg/L	14	10	0.095
≥ 0.04 μg/L	7	11	
PTH in the first month after surgery [pg/ml, M (Q1, Q3)]	32.10 (22.05, 46.50)	20.47 (9.43, 34.03)	0.192
Serum calcium in the first month after surgery (mmol/L, $\bar{x}$ ± s)	2.37 ± 0.06	2.29 ± 0.19	0.409
TSH in the six months after surgery [cases]			
≤ 2 uIU/ml	18	19	1.000
> 2 uIU/ml	3	2	
TSH in the first year after surgery [cases]			
≤ 2 uIU/ml	20	18	0.694
> 2 uIU/ml	1	3	
ns-Tg in the first year after surgery [cases]			
< 0.04 μg/L	19	18	0.583
≥ 0.04 μg/L	2	3	
Pathological subtype with poor prognosis [cases]			
No	21	19	0.206
Yes	0	2	
Postoperative complications [cases]			
Incision infection	0	1	N/A
Permanent hypoparathyroidism	0	1	

N/A, Not Available.

**Figure 6:**
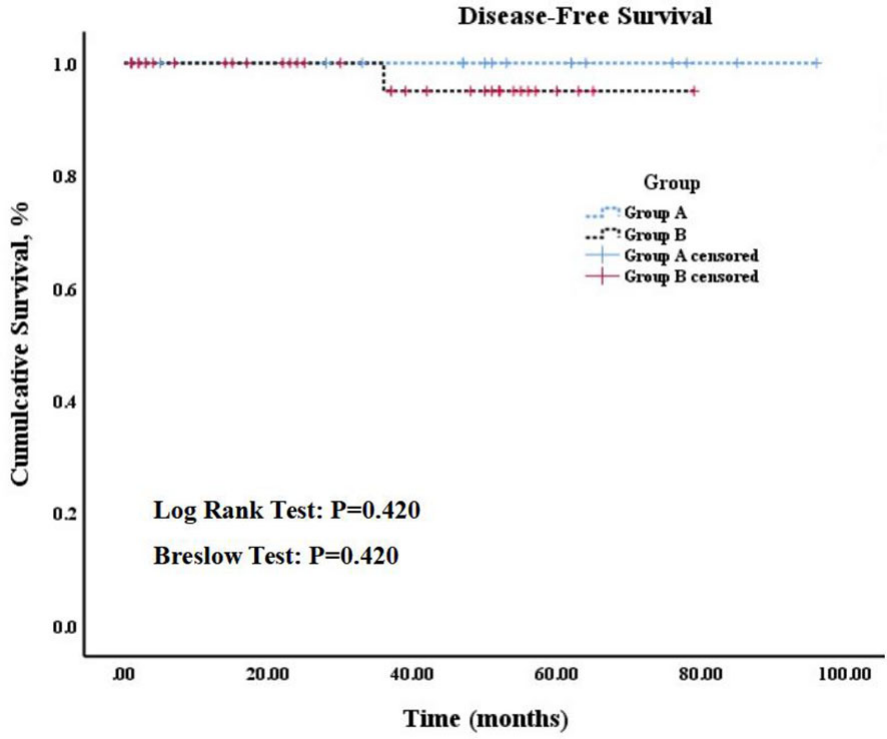
Kaplan-Meier survival curve.

The median follow-up period for the whole cohort was 44.5 mo (range, 5-96 mo), and no significant difference in follow-up time was observed between two groups (median, 44 mo [range, 5-96 mo] vs. 35 mo [range, 7-79 mo]; p = 0.339). According to the Kaplan-Meier curves, 5-year disease-free survival rates among patients in group A (100%) were not significantly higher than those among patients in group B (97.1%) (Log rank test: p = 0.420, Breslow test: p = 0.420). The details can be found in **Figure 6**.

## 4 Discussion

Our study revealed that PTC concomitant with HT was associated with the female sex, younger age, smaller tumor, a lower ratio of pathological subtype with poor prognosis, a lower central lymph node metastasis ratio, a better prognosis, but a tendency for multifocality, thus indicating the complexity of the effect of HT on PTC prognosis (12–14). HT interferes with the preoperative ultrasound evaluation for central lymph node (LNs) and increases the incidence of the absence of fatty hilum in central benign LNs, thereby increasing the incidence of misdiagnosis and unnecessary surgery (15, 16). For patients with thyroid cancer of >1 cm and <4 cm without extrathyroidal extension and clinical evidence of LN metastases (cN0), the initial surgical procedure can be either bilateral (neartotal or total thyroidectomy) or unilateral (10). The specific goals of initial therapy are to remove the primary tumor and clinically significant LN metastases and to minimize the risk of disease recurrence and metastatic spread. The utilization of intraoperative neuromonitoring and nano-carbon negative imaging technology has facilitated the integration of central LN dissection into our center’s standard surgical protocol. It is recommended that thyroidectomy without prophylactic central neck dissection is appropriate for small (T1 or T2) and noninvasive. Alternatively, for patients with some prognostic features associated with an increased risk of metastasis and recurrence (older or very young age, tumor subtypes with a poor prognosis, multifocal disease) or if the information will be used to plan further steps in therapy, prophylactic central-compartment neck dissection (ipsilateral or bilateral) should be considered (10). Therefore, the prophylactic cervical LN dissection was performed on all patients except for one. Although the incidence of cervical LN metastasis pathologically was relatively low in both the groups and no statistically significant difference was observed, this technology enables enhanced precision in disease staging and risk stratification and can be used to guide initial prognosis, disease management, and follow-up strategies, which is an essential component in the management of patients with DTC.

The ATA guideline (2015 Edition) recommends that in patients with an excellent (clinically and biochemically free of disease) or indeterminate response to therapy, especially those at low risk for recurrence, the serum TSH level may be kept within the low reference range (0.5-2 uIU/ml) (10). However, a recent meta-analysis study concluded that the evidence for the effectiveness of post-operative TSH maintenance level less than 2 uIU/ml in patients undergoing thyroid lobectomy for low-risk DTC is insufficient (17). The goal of TSH suppression therapy for patients with DTC stratified by low recurrence risk in our center’s clinical practice remains to maintain TSH levels below 2 uIU/ml, in accordance with the NCCN and ATA guidelines (5, 10). In our study, both groups of patients were classified as having low risk for postoperative recurrence, and their TSH suppression levels demonstrated satisfactory outcomes with the follow-up assessments.

Adequate surgery is the most important treatment variable influencing prognosis. The extent of surgery also plays important roles in determining the risk of surgical complications. Hypoparathyroidism is the most relevant complication in radical thyroid surgery and has devastating influence on the patients’ quality of life. Currently, permanent recurrent laryngeal nerve injury and postoperative hemorrhage rarely occur due to subtle surgical techniques (18). The texture of thyroid tissue in patients with HT differs from that of normal thyroid tissue, which lacks inflammatory cells or lymphocyte infiltration. In contrast to the soft texture of normal tissue, the affected tissue exhibits toughness. Additionally, if the associated cancer is located in challenging areas such as the upper or lower pole of the thyroid, near the recurrent laryngeal nerve, or in the isthmus, it can significantly increase surgical time and procedural difficulty. This finding has been supported by a retrospective cohort study (19). The drainage tube is routinely inserted during the operation at our center, and close observation of the color and volume of drainage fluid is conducted post-surgery. Furthermore, patients’ conditions are monitored for 1 day after extubation before discharge. Thus, the operation time and postoperative hospital stays of group A are evidently distinct from those of group B, indicating a significant difference between the two groups.

The recurrence rate of patients with DTC exhibiting excellent response to treatment is low (20). Limited evidence suggests a low utility of Tg level assessment in identifying recurrent or metastatic disease following partial thyroidectomy. Following total/near-total thyroidectomy, a cutoff Tg level of 1-2.5 ng/mL may help identify patients at low risk for persistent or metastatic disease (21). Most patients in both groups exhibited a ns-Tg level below 0.04 ng/mL at the 1-year postoperative mark. The emphasis should be placed on the fact that within group A, ns-Tg levels were lower than 0.04 ng/mL in 14 patients at one month after surgery and in 19 patients after the first year post-surgery, primarily attributed to standard postoperative TSH suppression therapy. The long-term follow-up results of both groups were also highly consistent with the degree of control of ns-Tg level.

Admittedly, this retrospective case cohort study was solely conducted within our center, and the sample size of included patients was relatively limited. During follow-ups, TG-Ab which is one of factors influencing postoperative Tg monitoring levels is not measured. Further multicenter prospective studies are warranted to validate the clinical utility of lobectomy with isthmusectomy in low-risk PTC patients with HT.

## 5 Conclusion

The combination of lobectomy with isthmusectomy and prophylactic central neck dissection represents a safe and viable treatment option for patients with low-risk PTC coexisting with HT. Proper and consistent TSH suppression therapy, along with regular monitoring, are crucial in preventing tumor recurrence.

## Data availability statement

The raw data supporting the conclusions of this article will be made available by the authors, without undue reservation.

## Ethics statement

The studies involving humans were approved by Biomedical Ethics Committee of Peking University International Hospital. The studies were conducted in accordance with the local legislation and institutional requirements. Written informed consent for participation was not required from the participants or the participants’ legal guardians/next of kin in accordance with the national legislation and institutional requirements.

## Author contributions

DZ: Writing – review & editing, Writing – original draft, Software, Resources, Formal analysis, Data curation, Conceptualization. JW: Writing – review & editing, Supervision, Project administration, Methodology. LC: Writing – review & editing, Visualization, Validation, Investigation.
